# Preventing and Treating Torsades de Pointes in the Mother, Fetus and Newborn in the Highest Risk Pregnancies with Inherited Arrhythmia Syndromes

**DOI:** 10.3390/jcm12103379

**Published:** 2023-05-10

**Authors:** Annette Wacker-Gussmann, Gretchen K. Eckstein, Janette F. Strasburger

**Affiliations:** 1 Department of Pediatric Cardiology and Congenital Heart Disease, German Heart Center Munich, 80636 Munich, Germany; 2 Division of Cardiology, Departments of Pediatrics and Biomedical Engineering, Children’s Wisconsin, Herma Heart Institute, Medical College of Wisconsin, Milwaukee, WI 53226, USAjstrasburger@chw.org (J.F.S.)

**Keywords:** fetal arrhythmia, Long QT, delivery room, Torsades de pointes

## Abstract

The number of women of childbearing age who have been diagnosed in childhood with ion channelopathy and effectively treated using beta blockers, cardiac sympathectomy, and life-saving cardiac pacemakers/defibrillators is increasing. Since many of these diseases are inherited as autosomal dominant, offspring have about a 50% risk of having the disease, though many will be only mildly impacted during fetal life. However, highly complex delivery room preparation is increasingly needed in pregnancies with inherited arrhythmia syndromes (IASs). However, specific Doppler techniques show meanwhile a better understanding of fetal electrophysiology. The advent of fetal magnetocardiography (FMCG) now allows the detection of fetal Torsades de Pointes (TdP) ventricular tachycardia and other LQT-associated arrhythmias (QTc prolongation, functional second AV block, T-wave alternans, sinus bradycardia, late-coupled ventricular ectopy and monomorphic VT) in susceptible fetuses during the second and third trimester. These types of arrhythmias can be due to either de novo or familial Long QT Syndrome (LQTS), Catecholaminergic Polymorphic Ventricular Tachycardia (CPVT), or other IAS. It is imperative that the multiple specialists involved in the antenatal, peripartum, and neonatal care of these women and their fetuses/infants have the optimal knowledge, training and equipment in order to care for these highly specialized pregnancies and deliveries. In this review, we outline the steps to recognize symptomatic LQTS in either the mother, fetus or both, along with suggestions for evaluation and management of the pregnancy, delivery, or post-partum period impacted by LQTS.

## 1. Introduction

Prenatal and delivery room care of adult women with Long QT Syndrome (LQTS) has been described [1,2,3,4,5,6,7,8], but less has been written about the assessment and management of Long QT Syndrome in the fetus presenting with severe LQTS-related arrhythmias [1,9]. It is possible to provide risk mitigation during pregnancy and delivery [8]. The focus of this review will be to provide practical guidance and resources to multidisciplinary teams involved in managing these high-risk pregnancies and the offspring of these pregnancies. The fetal arrhythmia manifestations will be reviewed along with the diagnostic modality needed to appreciate them.

Fundamentally, there are three main presentations—the mother who is affected with LQTS with (1) an affected fetus or (2) an unaffected fetus, or finally, (3) an unaffected mother who has an affected fetus with a familial variant of LQTS (father, sibling affected, whether living or deceased), or an affected fetus with a de novo variant of LQTS. Presentations vary with each; however, delivery room preparedness is similar.

While the pre-pregnancy and prenatal evaluations are not the primary focus of this review, they are included as part of the preparedness. It should also be recognized that most women with LQTS have successful pregnancies with little intervention; however, this should be after adequate precautions.

A. Mothers with symptoms on medications with or without ICD

It is important to risk-stratify the pregnancy. Mothers with LQTS or other ion channelopathy require ongoing assessment throughout pregnancy, in the delivery room, and especially in the post-partum period, up to nine months post-delivery [1,2,3,4,5,6,7,8].

Pre-partum: Ideally, for the couple contemplating pregnancy, where either the parent or a sibling or stillborn from a prior pregnancy is affected, a high-risk obstetrician and a geneticist for pre-pregnancy genetic counseling should be consulted.

At this consult, they would discuss important issues such as the risk of transmitting the familial disease. It is advisable that the mother undergo a pre-partum cardiology/electrophysiology examination to determine the status of her medications, defibrillator, and cardiac function. An exercise stress test can be helpful in determining the degree of beta-blocker effect on heart rate and blood pressure. A Holter might be needed if the patient is experiencing palpitations. Some ion channelopathies impact ventricular function, and therefore, a screening echocardiogram is advisable if not previously completed. In addition, if the implantable defibrillator is nearing elective replacement indicators, this should be addressed prior to pregnancy.

An emergency response plan between the patient and her electrophysiologist, her pacemaker clinic, and high-risk obstetrical caregivers should be established, and she should have emergency contact numbers with her or a MedicAlert bracelet. The electrophysiologist ideally should communicate directly with the obstetrical care team so adequate counseling can take place. Such counseling is outlined below in the “fourth-trimester” care of the family with LQTS. Ideally, the cardiac team should become part of the multidisciplinary obstetrical (OB) team meetings during the pregnancy. The patient should know which hospital to report to with certain symptoms. This is also when drug compliance and barriers to getting medications should be reviewed.

Ideally, these women will be managed through their pregnancies in obstetrical and fetal care centers of excellence; however, in many rural settings, this is not possible, and close collaboration between the local care team and the high-risk providers is necessary. Some families benefit from support groups such as the Sudden Arrhythmic Death Syndrome (SADS) Foundation, which provides multimedia education online, annual patient-based meetings, and newsletters (SADS.org). The patient may perceive herself as healthier, or if an ICD is in place, more invincible than she is, and may request to deliver at home or in low acuity settings. This should be addressed early by reviewing the risks that this presents. In general, if an ICD is functioning, the interrogation can occur on the usual schedule without extra visits (in the absence of syncope or palpitations).

Pregnancy is not usually the best time to transition medications in a woman who is stable [10,11]. At this pre-pregnancy consultation, discussions about the various medications and their impact on breastfeeding or growth restriction should be reviewed, with shared decision-making as to whether to alter therapy. Nadolol is the most commonly used and most effective beta-blocker (due to its long half-life). A recent study showed that while growth restriction was seen with beta-blockers [12], it was no more likely to be present with nadolol than with other drugs [13]. The best beta blocker for LQTS during pregnancy has not been proven. However, in one study, metoprolol was shown to be inferior in preventing events in LQT1 and 2 [14]. A subsequent study suggested similar efficacies [15], but in addition to this, Wilde and Ackerman [16] questioned the comparability of the two populations. Labetolol, a beta blocker that OBs may be familiar with for maternal pre-eclampsia treatment, has essentially no beneficial antiarrhythmic effects. Nadolol is passed more freely into breast milk than propranolol and can result in infant levels of about 5% of maternal levels, with peak concentration about 6 h after maternal ingestion [17]. Importantly, if transitions in any medications are planned, it is critical to see that the new prescription is for a long-acting or sustained-release form of beta blocker. Standard short-acting formulations of metoprolol or propranolol leave the patient with LQTS at risk for breakthrough arrhythmias. A sustained drug effect is needed.

Early Antepartum: Unlike standard pregnancies, it is advisable that the pregnant patient with LQTS have early care.. At a minimum, in-person visits at 12 weeks, 20 weeks, 28 weeks and 36 weeks gestational age are recommended [8]. More may be required to detect fetal growth restriction if the mother is taking beta blockers. Ideally, the mother will have presented prenatally, and the above medical/cardiac work-up has been completed, but if not, it can and should be done in the early stages of her pregnancy.

Hyperemesis gravidum is of concern for women with LQTS. It can cause electrolyte disturbances and weight loss that can potentiate the risk of arrhythmias. There should be an established plan for when to initiate intravenous fluid hydration or hospitalization for an alternative medication administration route.

It is important to monitor that the mother is taking adequate amounts of calcium and magnesium-rich foods (generally the equivalent of around 1200 mg of calcium and 360 mg of magnesium daily). Prenatal vitamins are generally a relatively poor source of both calcium and magnesium. Dietary handouts for foods rich in these nutrients are available from the National Health Institute (NIH) and other sources. Thyroid dysfunction and vitamin D deficiency are common in pregnancy and should be assessed (if not already done). Chronic stores of vitamin D support the absorption of calcium and magnesium from the gut and can be assessed with a 25 OH vitamin D level [18,19]. Unfortunately, many newer electronic health systems list the 1–25 OH vitamin D first (alphabetically), and this test only reflects recent intake and not chronic stores and should NOT be obtained.

Mid-to-Late Antepartum Care: At this stage of the pregnancy, medications may need to be adjusted to maintain adequate beta blocker effect during physiologic increases in cardiac output and major increases in circulating blood volume [17]. Some centers use exercise stress testing at 3–6 months gestation and again 3–6 months post-partum to assess the beta blocker effect, limiting the peak heart rate to no more than 60% of the predicted max for safety.

After 30 weeks of GA, mothers should have higher surveillance with biophysical profiles weekly or non-stress testing, as well as monthly fetal growth assessment if the mother is taking beta blockers [20,21]. Ideally, the ongoing high-risk pregnancy care plan should be reviewed regularly by the multidisciplinary management team, consisting of the referring adult cardiologist/EP specialist, the maternal-fetal medicine (MFM) team, the anesthesia provider (near term), the nursing support staff, neonatology, pediatric cardiology/EP, and the genetic counselor. Fetuses, even if the mother is not affected by the familial LQTS and even when not on a beta blocker, should have higher surveillance because of their 50% risk of LQTS in this setting.

Fetal assessment: For the fetus, the focus is on determining whether the fetus growth restriction, which is a common side effect of beta-blocker therapy and can be seen in up to 1/3 of exposed fetuses, yet it is generally relatively mild. For example, Cuneo et al. [21] showed that the average overall size of infants at birth where the mother was affected was about 2900 g whether or not the mother was taking a beta blocker, whereas the average weight where the father was the proband was about 3300 g. All fetuses with growth restrictions require a standard of care for this diagnosis, which is usually a screening at 20 weeks of gestational age, fetal echocardiogram and every 4-week obstetrical growth scan with weekly biophysical profiles after 30 weeks of gestational age [22]. If possible, providers should attempt to determine whether the fetus has an inherited arrhythmia syndrome. Fetal magnetocardiography, if available, is highly beneficial in risk stratification with over 90% sensitivity and specificity for accurate diagnosis of LQTS [20,23,24,25,26]. It is a non-invasive approach to diagnose fetal arrhythmias precisely. While fetal ECG has also been shown to diagnose LQTS in a small number of cases [27], FDA-approved software is not currently available and reliable in the 15–40 weeks of gestation range and is less than for fMCG.

Anticipatory Preparedness for Family and Staff: Everyone can take advantage of the pre-delivery timeframe to optimize preparedness for both family and obstetrical staff.

Genetic counselor considerations: The cardiogenomics company performed genetic testing on prior family members should be determined. Testing may be substantially less expensive if the same company can be utilized for the infant and for cascade testing of other family members. Having a kit in the delivery room can facilitate the acquisition of cord blood. An alternative is to use a buccal swab. The prepartum timeframe can be used for parental and caregiver CPR training as well since they may not be as busy then as during post-partum.

**Figure 1:**
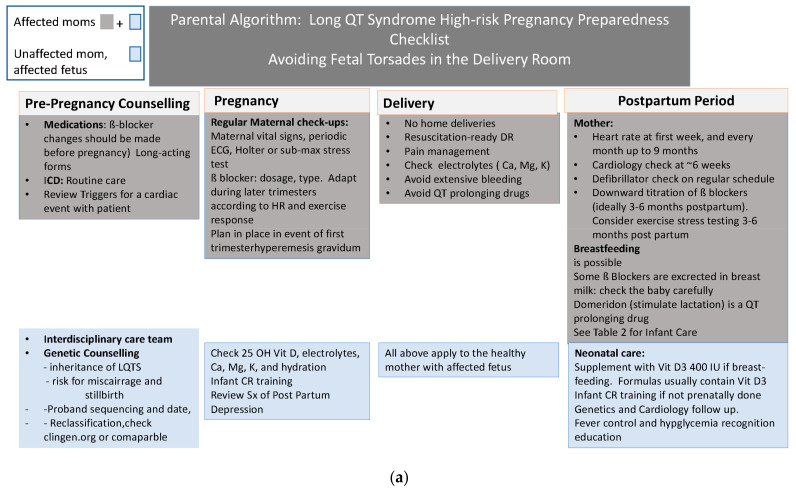

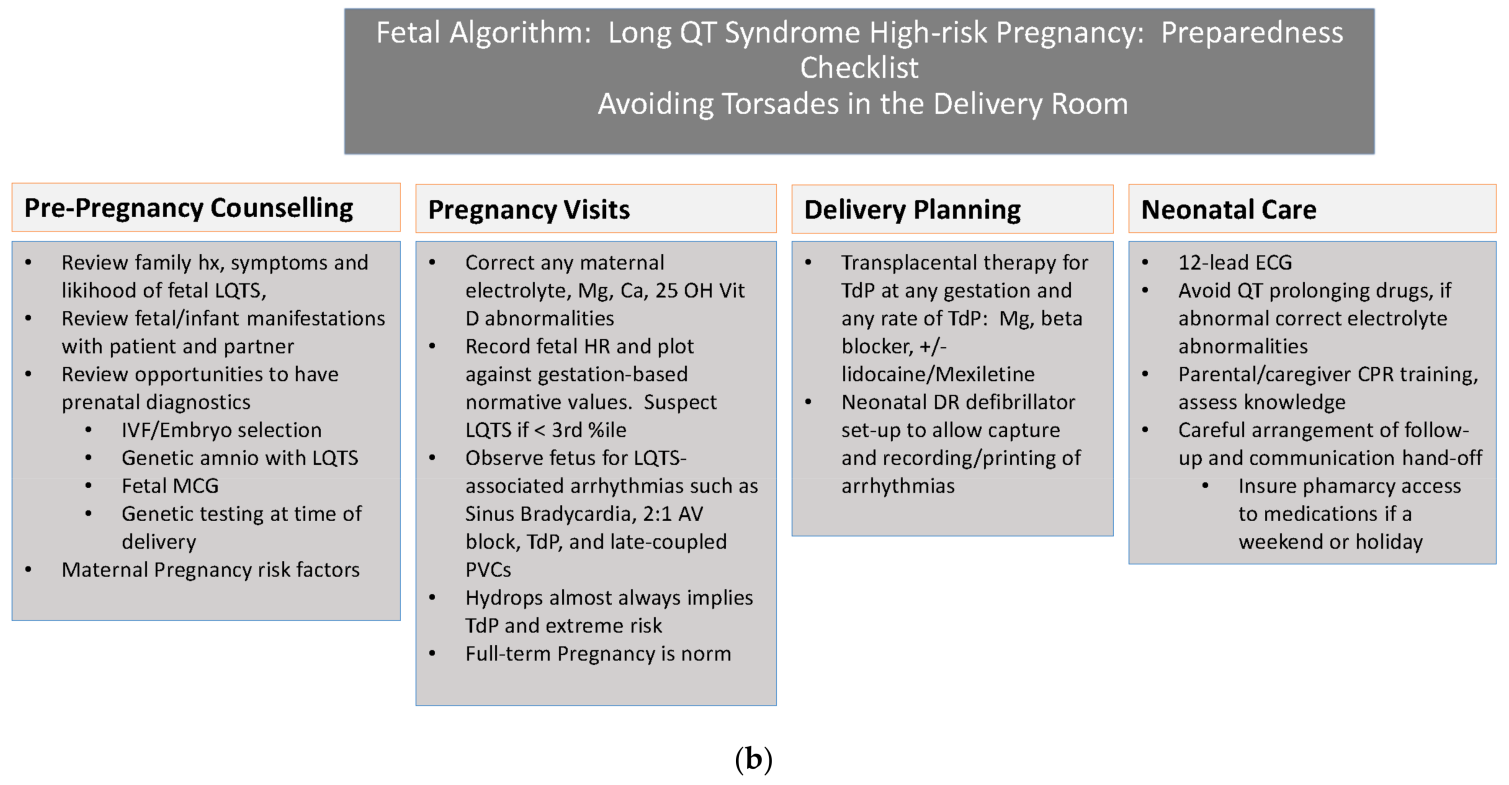
(**a**) Parental Algorithm Avoiding Fetal Torsades in the Delivery Room. (**b**) Fetal Algorithm.

Delivery Room: One of the most important places for preparedness is the delivery room. Figure 1 reviews the steps to prepare the delivery room and personnel for the delivery of a high-risk mother or fetus with LQTS.

For the unaffected mother and fetus with no symptoms, standard delivery is possible; however, for both the symptomatic mother and/or the symptomatic fetus, a scheduled induction at about 39 weeks gestational age when important team members can be available is advantageous.

It is advised that CPR modifications and defibrillator pad placement should be monitored specifically for late gestation pregnancies if the mother is affected [28].

If the patient has an ICD, it should be determined by the manufacturer as to what precautions need to be taken for the use of cautery.

Care should be discussed with anesthesia prior to the date of delivery. QTc-prolonging anesthesia medications, if at all possible (www.qtdrugs.org) should not be used. The affected pregnant women will need non-invasive cardiac monitoring during labor, but the use of invasive blood pressure monitoring is not necessary in most cases. A C-section is not advised for cardiac reasons.

Medication: Women with IAS should continue to take their cardiac medications during labor. It is important to be sure that NPO orders are clearly written so that these are not withheld. Magnesium is highly effective for Torsades de Pointes Ventricular Tachycardia (VT). Amiodarone (used in other cardiac arrests) is contraindicated during arrest in an IAS patient. Lidocaine, a short-acting beta blocker, and rarely isoproterenol or temporary pacing can be used. Very rarely, uterine evacuation for sustained cardiac arrest could be required. Reviewing plans for this with staff from the emergency department and Labor and Delivery during a mock code is ideal.

Care of the fetus exposed to maternal cardiac medications: It is important to review with the mother that she should stay on her medications and should not discontinue them because of pregnancy or breastfeeding. The transition to the commonly used obstetrical beta blocker, metoprolol, is controversial, may potentially provide inferior arrhythmia control, and is unlikely to be necessary [13,14].

Special consideration for the delivery mode of a miscarriage or for Stillborn infants: If the pregnancy is lost, it is important to consider LQTS testing. For the family recovering from fetal loss, it is important to have a stable contact person from nursing or genetics who keeps in touch with them. Post-partum depression is more common when a fetal loss occurs and can result in weight loss, medication non-compliance, and the inadvertent prescribing of drugs that lengthen QTc, such as antidepressants. Barriers to follow-up or mental health support should be assessed, especially if the length of contact with the OB team has been brief (early miscarriage). Beta-blockers have occasionally precipitated or contributed to depression. Communication with the adult EP and pacer nurse, if approved by the patient, is important so that patient support can come from many sources.

Post-Discharge Care for the Infant: Family members and babysitters providing care for the infant need knowledge about emergency response. CPR training is now online and can be started at any time in a family with this condition, preferentially prior to delivery. Likewise, the risk of the familial variant of LQTS should be considered in terms of a home automated external defibrillator (AED). If a device is available, current scientific statements support the use of an Automated Defibrillator Device in any affected family members, including an infant, if found unresponsive and without a pulse.

Care of the Newborn: Infants may be at risk for hypoglycemia if growth is restricted due to in utero exposure to beta-blockers or while breastfeeding, if there is maternal use. They may not respond as well to sympathomimetic medications.

B. The Fetus manifesting LQTS rhythms

Antepartum Manifestations: In a review of fetal and neonatal LQTS in Japan, 18% of cases were presented in utero, and nearly 85% in the first few days of life [29,30]. Hence, having preparedness when LQTS is anticipated, both in the delivery room and in the neonatal nursery, makes sense. The risk of having fetal LQTS or other IAS in a pregnancy where either parent or a sibling is affected is usually 50%, though higher if mother/daughter transmission [21]. Few conditions confer such a high risk of potential arrhythmias at such an early gestation. Based on the findings of Strand and colleagues [20], as well as Cuneo and colleagues, it is also likely that some cases go unrecognized until stillbirth [21] or miscarriage happens. Stillbirth is often incorrectly attributed to an unknown cause rather than to an ion channelopathy. Our recent manuscript showed that seven of nine fetuses with Torsades de Pointes (TdP) events were unrecognized as such by echocardiography.

In our recent series, 11% of familial LQTS fetuses evaluated for suspected LQTS-related arrhythmias or familial risk manifested TdP, but none died, whereas de novo cases had an 85% chance of having an LQTS-related arrhythmia, 55% risk of TdP, and a 44% rate of stillbirth or infant death. Hydrops fetalis, SSA-negative AV block, and unusual tachy-brady rhythms are common prenatal presentations in this group, and since TdP is usually not recognized by fetal echocardiography, fMCG plays an increasingly important role in risk stratification. Unfortunately, it remains confined to a few institutions.

Based on our recent studies, there are multiple LQTS-associated arrhythmias, and these will be described in the next section. Some are more severe than others. They are (1) sinus bradycardia, (2) 2:1 or 3:1 atrioventricular (AV) block, (3) late-coupled premature ventricular contractions (PVCs), (4) T wave or QRS alternans, sometimes also associated with mechanical alternans, (5) TdP with or without monomorphic ventricular tachycardia (VT), and finally combinations of the two or more rhythms in a “tachy-brady syndrome”, often combining second degree AV block with TdP or rarely atrial flutter.

Sinus bradycardia: The most common and earliest recognized presentation of the fetal arrhythmias associated with LQTS in pregnancy is sinus bradycardia. Winbo and colleagues [31] showed in a large family of LQT1 that the average heart rates declined during gestation in both normal and infants with a single gene variant but were lower than in controls [31]. The rates were even lower (111 +/− 6 per minute) for double mutations vs. 134 +/− 8 for single mutations. For those with neonatal QTCs over 550 ms, the heart rate fell in proportion to the increase in QTc. If there is a maternal history and if she is taking beta blockers, these can have about a 7–10 beat/min lowering of the fetal heart rate throughout gestation; however, this effect is modest and major changes, especially early on, in gestation-specific heart rates should raise suspicion for LQTS. The establishment of the fetal heart rate early by ultrasound, with subsequent gestation-based normative HR data at each visit, is important [23,26,32]. LQTS1 usually has consistent sinus bradycardia, whereas LQTS2 often does, and it is less reliable seen in other IASs [20]. Whether or not bradycardia is noted, the clinician should be cognizant of the echo/Doppler rhythm findings associated with severe LQTS [33]. Finally, even when a family Hx of LQTS is lacking, it is important to recognize that many cases of LQTS go completely unrecognized despite abnormally low fetal heart rates because obstetricians often discount the importance of heart rates around 110–135 beats/min as being normal variations. It is exceedingly rare to see normal fetuses with persistent rates below the third percentile, and preferably in the future, when FMCG is more available, these pregnancies will be referred. Until then, neonatal ECG is advised for persistent sinus bradycardia. There are very few other causes for these present bradycardias other than IAS, but some additional causes include low atrial rhythm due to heterotaxy syndromes and sinus node damage due to SSA isoimmunization [34,35]. Other hereditary bradycardia syndromes, such as HCN4 and NKX2.5, can also present with sinus bradycardia. The point being that rates persistently lower than the third percentile need to be assessed with fMCG or, at minimum, neonatal ECG to exclude IASs.

### 1.1. Second Degree AV Block, T Wave, QRS, and Mechanical Alternans and Torsades de Pointes Tachycardia

When the QTc is markedly prolonged, 2:1 and even 3:1 atrioventricular block develops and is sometimes transient. When it occurs suddenly, fMCG often documents a sudden and dramatic lengthening of the QTc during the lower rate, which may place the fetus at risk for TdP. 2:1 and 3:1 AV blocks are often first attributed to isoimmune AV block, but in LQTS, the SSA/SSB antibodies are negative. This type of block has been seen with Timothy Syndrome; therefore, a quick review of the ultrasound looking for findings of micrognathia, growth restriction, and syndactyly of the second and third digits of the hands or toes can provide a clue to the diagnosis. An AV block was common and was seen in 6–12% of LQT1 and 2 and over 50% of LQT3, compound, rare variants, and untested [20]. During the 2:1 block, the usual ventricular rate is less than 75 bpm, and during the 3:1 block, it can be as low as 50 bpm, making it easy to confuse with a third-degree AV block. The goal in the management of functional AV block is to shorten the QT interval; therefore, magnesium, beta-blockers, lidocaine, mexiletine, calcium, vitamin D (if 25 OH Vit D is low), and potassium may provide benefit, along with avoidance of or withdrawal of QT-prolonging drugs such as those listed at qtdrugs.org or the SADS website [24,25,36]. Hydrops has been seen with second-degree AV block, but when present, it is due to TdP rather than bradyarrhythmia. Terbutaline, which has been used to speed ventricular rate in case of isoimmune AV block, has been very proarrhythmic, exacerbating TdP in one case (Strasburger, JF Personal experience). Fetal MCG has proven highly useful in this setting.

When the family history is negative, this type of block, when not caused by autoantibodies (SSA, SSB), is often seen in de novo LQTS. Other hints for de novo LQTS may be beat-to-beat alternating ventricular outflow velocities (mechanical alternans [20] seen with QRS or T wave alternans, late occurring PVCs, and TdP). Unlike other forms of monomorphic VT, this rapid polymorphic VT is associated with severe diminution of cardiac output, resulting in little forward flow during many semi-lunar valve openings (hence, it does not appear as a tachycardia by Doppler), making it difficult to be recognized by the echo that a rapid VT is occurring [34].

Evaluating valve clicks and the timing of ejection can be helpful in identifying the chaotic nature of rapid TdP. In addition, rapid TdP is often accompanied by normal rate TdP as well as slower monomorphic VT, possibly masquerading as SVT with 1:1 conduction [34]. Similarly, atrial flutter has recently been identified with TdP in the fetus and similarly re-directs the clinician to suspect diseases other than LQTS [34].

Unexplained hydrops fetalis often accompanies this presentation. Early referral for fMCG can be life-saving [1,20,37], as one-third of those with AV block develop TdP, and nearly half of those with TdP go on to die unless aggressive treatment is initiated. In our experience (JFS), 9 of 11 fetuses with TdP at the time of fMCG had not been suspected of having TdP prior to the fMCG [4].

Echo rhythm assessment should include isovolumic relaxation time and mechanical PR and RR interval, as well as inferior vena cava/ductus venosus flow, superior vena cava/aortic flow, and a complete echo/Doppler with M-mode through the atrium and ventricle simultaneously [33]. Like sinus bradycardia, all other categories of LQTS-related arrhythmias require full fetal echo/Doppler evaluation by skilled fetal cardiologists.

Torsades de Pointe Tachycardia: Once Torsades is identified by echo or fMCG, a number of management strategies can be instituted. First, genetic testing of amniotic fluid might be possible in order to identify whether the fetus has a severe de novo or an unrecognized familial LQTS. Amniotic fluid cells are primarily fetal in origin. If prenatal testing would not alter treatment, postnatal evaluation is adequate. Both parents could be tested prenatally, if possible, by ECG and genetic testing. Low maternal magnesium, calcium, potassium, and vitamin D levels should be managed. Our anecdotal experience is that the use of up to 50,000 U of vitamin D per week may help replenish low vitamin D stores. For transplacental therapy, the mother should be loaded with intravenous magnesium before any contemplation of delivery. She can be maintained with magnesium and oral propranolol provided that she does not have severe asthma and transitioned to a sustained release beta blocker, or if she is full term, to delivery [24,38,39,40]. In urgent cases, a short-acting intravenous beta-blocker, such as esmolol, can be used. Propranolol appears to have the best transplacental transfer [17]. Depending on the type of LQTS, other drugs may be considered, such as lidocaine or mexiletine [41]. Relatively high doses of these medications may be required in order to control the tachycardia.

While based on a limited number of cases, transplacental management of TdP may be reasonable prior to delivery even when detected at a gestation ≥36 weeks. Such an approach has been utilized recently with good success for SVT. (Cuneo, personal communication). Transplacental treatment can allow for some resolution of hydrops and can provide a window during which postnatal transitional treatment can be implemented prior to any TdP. Unlike supraventricular tachycardia, TdP is very poorly tolerated hemodynamically in the infant and often leads to cardiac arrest. Therefore, prevention is vital.

### 1.2. Antepartum Evaluation and Management

FMCG has been shown to be effective in diagnosing LQTS at any gestation over 18 weeks, but if the fMCG is normal under 27 weeks GA, it should be repeated in order to capture those cases of LQTS that present late [34]. Finally, fMCG is invaluable in defining the progression of LQTS-related arrhythmias over the maturing pregnancy, as well as for serial assessment during antiarrhythmic therapy. Our center has documented unusual responses of these fetal diseases to treatment, further highlighting the fact that the type of drug treatments in utero may not be the same as in older children [34].

### 1.3. Delivery Room Management of the Severely Affected Fetus with LQTS or Suspicious Rhythm

First, many fetuses with LQTS do not have severe manifestations at birth. In those cases where fMCG has shown no QTc over 590 ms, management can be simplified. However, preparing for the highest-risk delivery is important when either de novo or severe familial LQTS is present. In the days approaching the delivery, our regimen is to optimize maternal magnesium levels through nutrition or oral supplementation, and if TdP has been previously encountered in either mother or fetus, a bolus of 1–2 gm/kg of intravenous magnesium sulfate on admission is advisable. Other maternal management recommendations have been outlined above. Commonly prescribed drugs during labor and delivery, such as Pitocin (oxytocin), azithromycin, and ondansetron can prolong the QT interval, as can opioids used for pain management. Similarly, epinephrine-containing agents for epidurals, as well as certain local anesthetics transfer readily to the fetus and can be arrhythmogenic. The fetus does not necessarily have to be delivered by C-section, but the risk-benefit of C-section should be considered in the overall picture of the mother and fetus. Having a timed delivery with adequate staffing is ideal.

For the severely affected fetus with TdP and/or a second AV block, the delivery room setup is critical. It is one of the few times when a pediatric cardiologist or electrophysiologist may be needed. It is often helpful prophylactically to temporarily transvenous or epicardial pace neonates with LQTS-related AV block during initiation of antiarrhythmic therapy as lower heart rates often trigger TdP. Esmolol 50–200 mcg/kg/min intravenously can be used, followed by oral propranolol or nadolol. Isoproterenol can be mixed for the delivery room to augment heart rate as needed, and in a cardiac arrest situation in the delivery room, external pacing of the infant using the defibrillator can potentially support circulation temporarily. These deliveries are best planned for a time when back up in the Cath Lab (for emergency temporary ventricular pacing lead placement) or Operating room (for cardiovascular surgery to implant a pacing device, or permanent subcutaneous leads, or to establish extracorporal membrane oxygenation (ECMO) may be utilized.

Newborn Care: The majority of infants born to families with LQTS do not require special delivery room management. They should be monitored in a neonatal ICU until a 12-lead electrocardiogram can be obtained. If abnormal, they should have a pediatric cardiology consult and remain monitored [42].

About half of the infants with LQT1 show shortening of their QTc, and almost all other types of LQTS will show significant shortening of their QTC after birth [20,43]. It is unclear why this occurs. Moore and colleagues [43] showed that the prognosis for those fetuses with familial and de novo variants of LQTS is reasonable even with a severe fetal or neonatal presentation. The exception to this in their series of 81 patients were the infants with SCN5A. These infants were much more likely to have cardiac arrest events or death and had lower event-free and total survival by age ten years, leading these authors to suggest that cardiac transplant could be a consideration for SCN5A or variants of SCN5 A (linked to Long QT 3) [43]. First, however, left stellate ganglionectomy and ICD therapy are warranted. Recently, phenotoin has also been suggested as a treatment for certain genotypes where mexiletine and beta blockers have failed.

If the mother is breastfeeding, her medication list should be screened for drugs that lengthen the QTc. www.qtdrugs.org. These should be reviewed at each pediatric visit and especially if she develops post-partum depression since most SSRI antidepressants lengthen QTc. Post-partum depression in the mother can sometimes lead to short-term memory issues that can potentially impact the dependability of her medication administration for both mother and infant.

Finally, the planned management of TdP in the delivery room can be an opportunity for nursing education. One study showed that despite ACLS training, L and D resuscitations were problematic, and more recent studies have focused on training techniques [42,44,45]. Obstetrical and neonatal nursing teams can work with their hospital emergency preparedness (code) teams to provide mock codes appropriate to maternal or fetal arrhythmias and to review available equipment to be sure that it is sufficient for both a maternal cardiac arrest or a neonatal cardiac arrest due to LQTS. A recent study from two decades of LQTS pregnancy care at the Cleveland Clinic showed that only five cardiac syncopal events or cardiac arrests occurred during that time and that none were pre-partum or during labor or delivery, but instead, all were in the post-partum period up to 9 months post-delivery, and all were in women who were not aware of their disease at the time [13]. Four of five were in women with LQT2, which is known to be associated with a higher risk for post-partum cardiac arrest. Thus, close follow-up is required for women with LQTS in the post-partum period with a focus on helping them achieve adequate sleep, lower stress levels, and avoid provocative triggers. Nadolol appears to be the best drug for LQT2. It is encouraging that no women who were adequately treated with beta-blockers or ICDs had symptoms.

## 2. Conclusions

In summary, the pregnancies in those patients with LQTS or other inherited arrhythmia syndromes require special preparedness and a high level of understanding in order to optimize care. Such care should extend at least 9 months post-partum, as the post-partum period is the highest risk period. When a family history of LQTS is present, fetuses are at high risk of having the disease, whether the mother, father or sibling is affected. Fetal Torsades is often not detected by echocardiography, and when available, fetal MCG can be very useful in risk-stratifying the pregnancy with IAS. However, even in the absence of fMCG, it is possible to utilize echocardiography in specific ways to better detect LQTS-related arrhythmias.

## Abbreviations

Long QT Syndrome (LQTS), Torsades de Pointes Tachycardia (TdP), Ventricular Tachycardia (VT), Implantable Cardioverter Defibrillator (ICD), electrophysiologist (EPS) Obstetrician (OB), Maternal—Fetal- Medicine (MFM), Gestational Age (GA), Food and Drug Administration (FDA), Electrocardiogram (ECG), Cardiopulmonary Resuscitation (CPR), atrioventricular (AV), premature ventricular contraction (PVC), fetal magnetocardiography (FMCG), Catecholaminergic Polymorphic Ventricular Tachycardia (CPVT), and inherited arrhythmia syndromes (IASs).
